# Bending-driven patterning of solid inclusions in lipid membranes: Colloidal assembly and transitions in elastic 2D fluids

**DOI:** 10.1093/pnasnexus/pgae331

**Published:** 2024-08-07

**Authors:** Weiyue Xin, Maria M Santore

**Affiliations:** Department of Chemical Engineering, University of Massachusetts Amherst, Amherst, MA 01003, USA; Department of Polymer Science and Engineering, University of Massachusetts Amherst, Amherst, MA 01003, USA

**Keywords:** 2D colloidal assembly, chaining, lattice, elasticity, curvature

## Abstract

Biological or biomimetic membranes are examples within the larger material class of flexible ultrathin lamellae and contoured fluid sheets that require work or energy to impose bending deformations. Bending elasticity also dictates the interactions and assembly of integrated phases or molecular clusters within fluid lamellae, for instance enabling critical cell functions in biomembranes. More broadly, lamella and other thin fluids that integrate dispersed objects, inclusions, and phases behave as contoured 2D colloidal suspensions governed by elastic interactions. To elucidate the breadth of interactions and assembled patterns accessible through elastic interactions, we consider the bending elasticity-driven assembly of 1–10 μm solid plate-shaped Brownian domains (the 2D colloids), integrated into a fluid phospholipid membrane (the 2D fluid). Here, the fluid membranes of giant unilamellar vesicles, 20–50 μm in diameter, each contain 4–100 monodisperse plate-domains at an overall solid area fraction of 17 ± 3%. Three types of reversible plate arrangements are found: persistent vesicle-encompassing quasi-hexagonal lattices, persistent closely associated chains or concentrated lattices, and a dynamic disordered state. The interdomain distances evidence combined attractive and repulsive elastic interactions up to 10 μm, far exceeding the ranges of physio-chemical interactions. Bending contributions are controlled through membrane slack (excess area) producing, for a fixed composition, a sharp cooperative multibody transition in plate arrangement, while domain size and number contribute intricacy.

**Significance Statement**

The assembly of solid domains and inclusions within otherwise fluid sheets and flexible lamellae affords a means to dynamically pattern ultrathin materials into beautiful structures with potential utility in flexible optics and electronics, and functional skins. Directing this patterning through the elasticity of the sheet itself, exploiting bending-mediated attractions and repulsions, constitutes an unexplored avenue to control material structure, while understanding of bending-mediated interactions provides insights into biological membranes. The sharp, fundamentally important patterning transition demonstrated here holds potential utility in sensing applications. With the interactions of elastic origin having the ranges of up to tens of microns, far exceeding interactions of physio-chemical origins, patterns derived from elastic interactions may sustain further chemical functionalization of the inclusions themselves, further enhancing the functionality options.

## Introduction

The membranes of biological cells, which integrate proteins and other macromolecules into a fluid phospholipid bilayer, are a prominent example within a broader class of ultrathin composite materials. The material physics of ultrathin composite sheets transcends biology (1), and may someday enable sophisticated applications, for instance color changing skins, touch sensors, and reassembling circuit-containing films. Distinct and potentially useful behaviors achieved by ultrathin fluid–solid composites include the ability to assume complex contours and to assemble integrated molecules (e.g. proteins), phases (e.g. lipid rafts), and other objects (e.g. viral capsids) (2–5). The kinds of assemblies possible at certain compositions and conditions, the underlying interactions, likely go beyond by what has been observed with cells to include other membrane objects. Here, we focus on a biomolecular system of a phospholipid fluid membrane fluid integrating rigid phospholipid microplates: we identify regimes of microplate assemblies and report a potential cooperative transition in assembly.

The behavior of solid micron-scale plates in a fluid membrane can be conceived as a 2D colloidal suspension. Because many phenomena (diffusion, flow, and aspects of stretching and bending) within lamellae are well-described by features only in their extensive lateral direction (6), lamellae are often conceptualized as 2D. 2D does not mean always flat like a supported bilayer or a monolayer on a trough. Rather, the lamellae of the current work are ultrathin fluid sheets that dynamically assume complex contours with nonzero Gaussian curvature, i.e. not confined to a flat or even cylindrical shape. Further, in this study, the discrete Brownian character of the solids integrated into these fluid sheets enables their description as a 2D colloidal suspension. In addition to the potential influence by the usual colloidal interactions, the assembly of colloidal objects in membranes, i.e. 2D suspensions, is governed by interactions originating in-membrane elasticity.

Studies targeting an understanding of living cells often exploit giant unilamellar phospholipid vesicles to capture the essential membrane physics. These behaviors provide a point of comparison for the current 2D colloidal suspensions. Studies with phospholipid GUVs have revealed how functional interactions are regulated by the local membrane curvature produced by embedded or adhered objects, macromolecules, and lipid rafts themselves (7). For instance, though line tension drives the coalescence of discrete fluid membrane domains (8, 9), line tension can also distort fluid domains to produce stabilizing interdomain repulsions (10–12). This line-tension driven instability, causing domains to bulge, occurs only when domains are large enough and vesicles sufficiently under-inflated. The resulting long-range repulsion can give rise to domain ordering, for instance producing raspberry-like lattices of bulging domains (11).

In contrast with the repulsive interactions of fluid domains, adhesive nanoparticles (13–15) and membrane proteins (7, 16–18) act as attractive inclusions. Beyond the physico-chemical influence from molecular disruption near the point of adhesion, the membrane curvature resulting from partial engulfment produces longer range attractions that concentrate small objects (7, 19). Adherent nanoparticles can form plaques with closely associated hexagonal order or belts of more distantly associated inclusions that encircle the waist of under-inflated vesicles (13). A competition between adhesion and membrane bending has been suggested to govern the regimes where these different structures are seen. Separately, simulations of NBar proteins reveal string-like structures where protein features arrange regularly along the strings, implicating the detailed protein structure in the assembly morphology (20).

Flat membrane objects comprise yet a different type of inclusion. In this category, solid phosphatidylcholine membrane domains possess molecular order and symmetry across the bilayers (21–24), tending to be flat (25, 26) and rigid against shear (6, 26). Thus, discrete solid domains behave as plate-shaped colloids within otherwise fluid phospholipid membranes. In these 2D fluid–solid composites, both the solid plates (2D particles) and the fluid membrane can bend and undergo limited stretching. However, unlike the plates, the fluid can shear in-plane, allowing it to take on Gaussian curvature (6). We recently demonstrated how the resulting pairwise inter-plate interactions, with two in each vesicle membrane, contained both attractions and repulsions that enabled the plates to rest at a fixed separation with only submicron fluctuations in their relative positions along the membrane contour (25). The excess membrane area, defined as the actual vesicle area normalized on the area of a sphere of equal volume, was the key parameter in determining the relative plate positions in these 2-colloid systems. Further by tuning excess area through osmotic inflation or micropipette manipulation, the domains could be reversibly repositioned between relatively stable states. A simple model demonstrated that this behavior could be reproduced with fluid bending as they key material property and not requiring a line tension or physico-chemical interactions of the domains. The treatment predicted that the impact of excess area would decrease with small domain sizes or area fractions. Thus, it was established that plate-shaped colloids experience tunable mechanical interactions that differ from exclusively fluid domains or nanometric inclusions in a membrane.

The current work examines multibody assemblies in 2D elastic suspensions of colloidal plates and highlights morphologies that cannot be anticipated from the current understanding of pairwise interactions. Directed by fluid membrane bending, but different from the instability-induced repulsive lattices of fluid domains, the plates appear to maintain zero Gaussian curvature while arranging into ordered lattices, chains, or ordered plaques suggestive of combined attractions and repulsions sometimes with anisotropic character. This variety of structures is explored for systems near a single composition, with a 17 ± 3% solid area fraction. Variations in colloid size and number change, over an order of magnitude, the intricacy but not the underlying character of the assembled patterns. We introduce the excess area, which regulates the bending energy of the system and dominates the state space, switching interactions from attractive to repulsive. In contrast with the gradual shifting seen with two colloidal plates (25), the multiplate systems assemble sharply and cooperatively with a small difference in excess area. Thus a variety assembled configurations, from repulsive lattices to chains of associated plates, can be formed using a single material composition.

## Results

Compositionally uniform giant unilamellar vesicles were produced by electroformation from films of lipid mixtures at elevated temperatures in the one phase region of the phase diagram. After room temperature storage, vesicle suspensions were reheated and cooled at controlled rates to produce vesicle membranes containing relatively monodisperse compact solid domains. With the solid domains visualized by their exclusion of a fluorescent tracer lipid, the solid area fraction, *ϕ* measured from fluorescence micrographs was found to be nearly the same for all vesicles, 0.17 ± 0.03. For an overall lipid composition of 40 mol% DPPC (1,2-dipalmitoyl-sn-glycero-3-phosphocholine), 60 mol% DOPC (1,2-dioleoyl-*sn*-glycero-3-phosphocholine), calculations based on the phase diagram and physical property data, in the Supplementary material, predict a solid area fraction consistent with these observations. At room temperature, the solid domains were stable against coalescence, maintained a fixed shape, and appeared flat or nearly so, consistent with prior work (25). For vesicles 10–40 μm in diameter, the numbers of domains per vesicle, 4–100, was controlled via the cooling rate. With a fixed solid area fraction set by the overall lipid composition, the domain number *N* per vesicle of diameter *D*_v_ is related to domain size via


(1) $$\;D_{d}/D_{v}=\;\sqrt{\frac{4\phi }{N}}.$$

In this work, *D*_d_/*D*_v_ varied in the range 0.05–0.45. Because the solid micron-scale domains are thin, flat, and exhibit Brownian motion when not strongly interacting, they behave as 2D colloidal particles. We therefore refer to them as colloidal plates.

### Arrangements of colloids in membranes

**Fig. 1.**
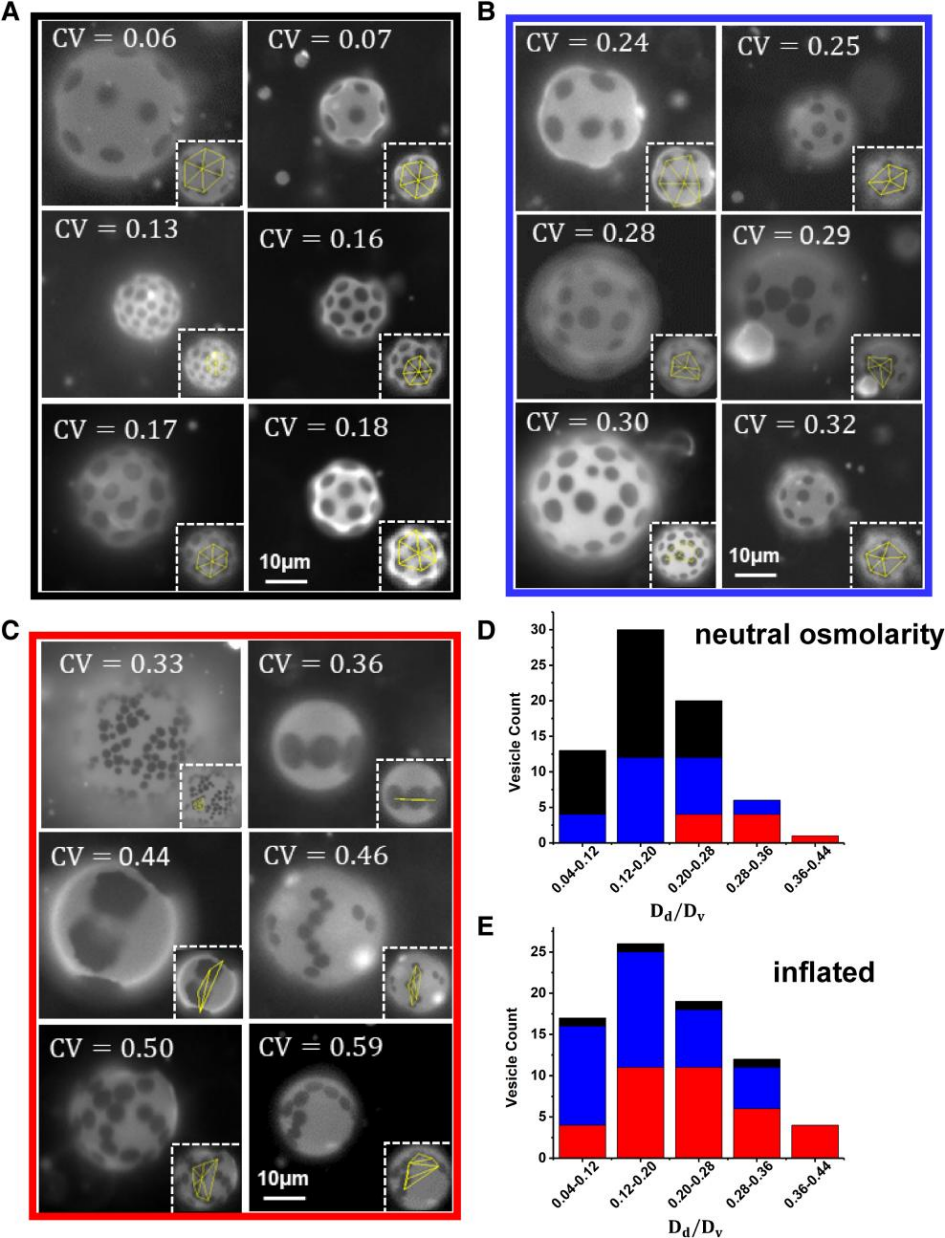
Examples of six vesicles for each of three pattern classifications: A) Vesicle-encompassing hexagonal quasi-lattice (black); B) Dynamic disordered state (blue); and C) Closely associated configurations (red). Each inset shows the limited Delaunay triangulation, featured at larger magnification in the Supplementary material. The histograms in (D) and (E) show the distributions of different pattern classifications found at D) neutral (100 mOsm inside and outside) and E) inflated (100 mOsm inside and 50 mOsm outside) osmotic conditions.

Figure 1 presents examples of the three types of patterns of colloids (solid Brownian membrane domains) observed in these systems: persistent vesicle-encompassing hexagonal quasi-lattices, persistent closely associated configurations (chains or concentrated lattices), and a dynamic disordered state. In the closely associated state, domains appeared not to touch. Configurations were classified as persistent if the colloidal plates visually exhibited limited translation on the timescale of minutes. In both persistent assembly types, Brownian motion is suppressed even though the plates are far from touching. This suggests substantial long-range interactions through the fluid portion of the membrane. Not surprisingly, then, persistent assemblies were not permanent and could be disrupted with gentle touch, later reforming similar but not identical structures. In the dynamic disordered state, domains translated on length scales of their own diameters within several minutes. The insets of each micrograph show the Delaunay triangulation analysis discussed below, with close-ups in the Supplementary material.

The vesicles exhibiting any of three classifications of colloid arrangements could be found within single electroforming and/or reheating batches, and we observed no obvious influence of vesicle size on the category of pattern found. Osmotic conditions, however, were found to be important in determining which category of pattern was observed. Figure 1D, summarizes the distribution of patterns found at neutral osmotic conditions, 100 mOsm both inside and outside vesicles. Here, vesicles of many sizes presented vesicle-encompassing hexagonal quasi-lattices or the dynamic disordered state. By contrast in Fig. 1E, when the vesicle suspension was diluted 50% in DI water so that the external osmolarity was reduced to 50 mOsm, conditions which tended to inflate vesicles, and the distribution of patterns was shifted to favor closely associated configurations or the dynamic disordered state. Thus, vesicles of the same material composition and batch were osmotically configured, to shift inter-plate colloidal interactions and the resulting assemblies.

While the types of arrangements are discernable by-eye, their differences were also systematically distinguished via image analysis. We employed a limited Delaunay triangulation method, accounting for the fact that not all plates could be in focus at once, detailed in the Supplementary material. Focusing on the top or bottom of a vesicle, the most central plate and up to six neighbors was identified. Neighbors were not necessarily “nearest neighbors” and fewer than six neighbors were selected only when there were fewer plate–colloids in the focal plane. Plate centers were connected by Delaunay triangulation, as shown in the inset of each image of Fig. 1A–C. The lengths of the center–center lines were averaged, and the standard deviation, divided by this average defines the coefficient of variation (CV).

In the examples in Fig. 1A–C, and for 151 vesicles in total, the CV values for each vesicle fell into distinct clusters, consistent with the initial visual assessment of pattern appearance. For instance for the vesicle-encompassing quasi-lattice configurations, CV < 0.2; for the vesicle with disordered dynamic positions, 0.23 < CV < 0.33; and for the closely associated structures, CV >0.34. Therefore, the limited Delaunay approach to pattern classification, which upholds the by-eye classification, established a lack of observer bias.

The limited Delaunay approach to pattern classification, used here only to confirm our by-eye classification, apparently works well because the CV is a measure of the variability in the spacing between the domains analyzed, adhering to the domain selection rules above. With human perception sensitive to pattern regularity, we see much more in the patterns than domain separation and yet the limited Delaunay methods does not consider additional factors such as angles between domains or numbers of nearest neighbors. Thus, the limited Delaunay method is, apparently, insensitive to certain types of defects, for instance, the occasional five rather than six nearest neighbors that occur when wrapping a hexagonal grid over a sphere. This is evident in the error analysis in the Supplementary material, which considers an example vesicle with a vesicle-encompassing hexagonal quasi-lattice with a prominent five-neighbor six-neighbor defect in the focal plane. If the center of the domain with six neighbors is selected, CV is 0.15–0.16. However, if the domain having five nearest neighbors is selected, CV is 0.14. This difference of 0.01 in CV is smaller than the variations one achieves (increasing CV up to 0.04) by moving a single domain away from its observed position. Measurement error in CV, focusing on the choice of domains toward the image center, typically contributes far less than 12% error due to vesicle curvature, calculated in the Supplementary material. Some inaccuracy is also incurred by how the FIJI program chooses the plate centers. Such error falls within the analysis in the Supplementary material that considered plates in altered positions. Such altered positions could result from a real plate change in position, for instance during “melting” of inter-plate structure, or could be introduce through image analysis error, typically not contributing more than CV variations of 0.01 (typically an increase not a decrease). Thus, the regularity in domain spacing, measured by the Delaunay triangulation method, is one of several features of a hexagonal lattice which is sufficient in the current work to classify domains, with the other patterns studied having substantially greater CV values, which also distinguish those patterns. While further information may be gathered by a deeper analysis, such study is beyond the current focus.

**Fig. 2.**
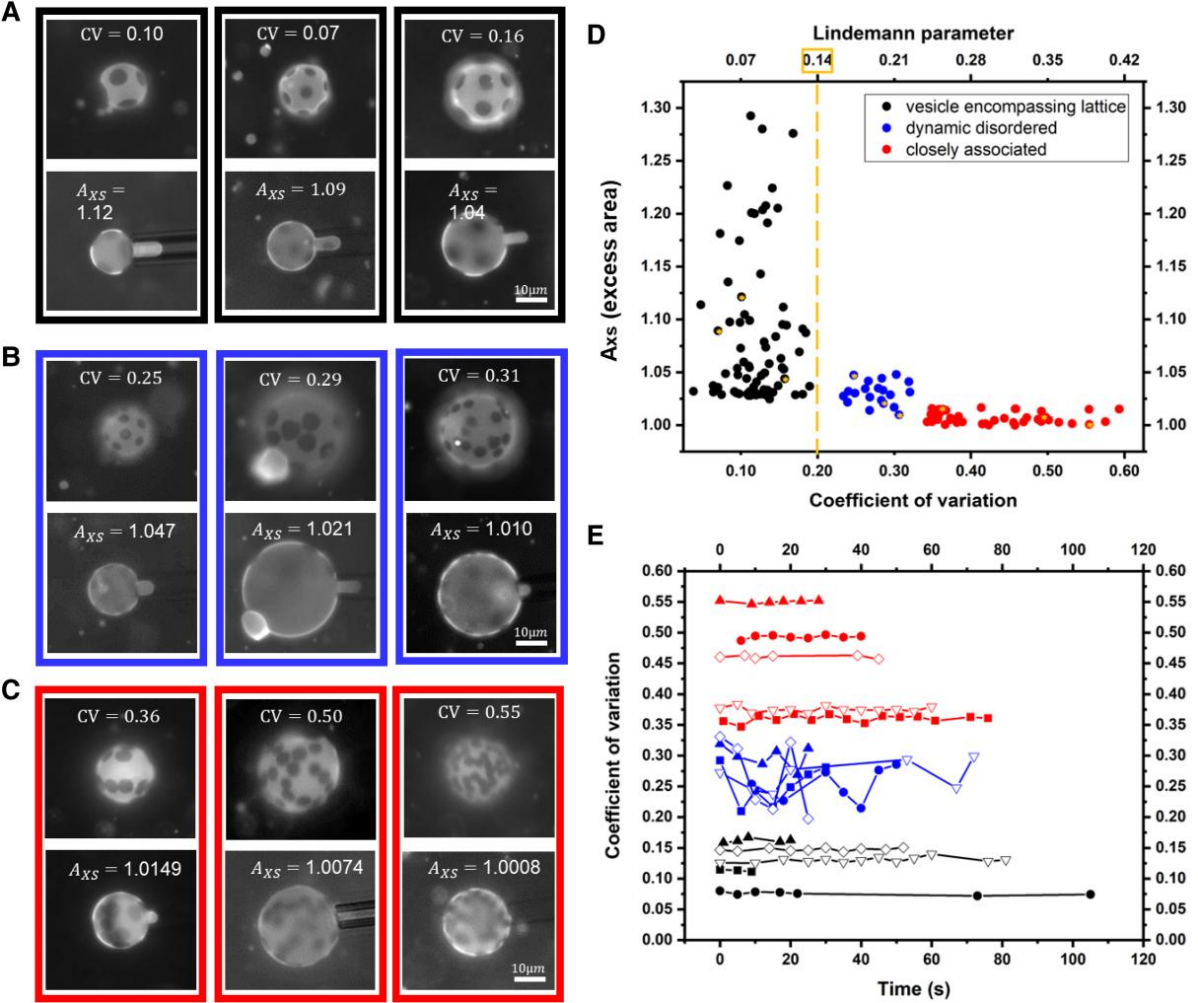
A–C) Examples of micropipette aspiration of vesicles at low-suction to determine excess area. Three vesicles are shown for each of the different pattern types: A) vesicle-encompassing quasi-lattice; B) dynamic disordered state; and C) closely associated configurations. The images show the greatest membrane projections into the pipette for the vesicle-encompassing lattice pattern. The scale bars apply to all images. D) The relationship between the excess area and the coefficient of variation from the limited Delaunay triangulation measured for each vesicle. The error in measuring domain positions, producing error in the reported CVs, as described in the Supplementary material, would contribute error in CV, increasing its upward by an amount of 0.02 for the black and blue data points. For the red data points, curvature effects and other measurement error contribute no more than an increase of 0.03 to the CV values in the worst case. Vesicles featured in (A–C) are starred in (D). E) Time variation in the measured CVs for each category of pattern. The solid symbols indicate vesicles which are featured in parts (A–C). Typically, vesicles were viewed for a few seconds at different focal planes to ensure a lack of tethers before being selected for micropipette study, and so for some vesicles, the video time for a given focal plane was limited. Therefore, two additional vesicles of each pattern type, represented by hollow symbols, were included also, in some cases interruptions in measurements corresponded to different focal planes or optical adjustments; however, the persistent patterns typically are unaltered on the timescale of minutes.

It is, however, worth noting that the dynamic Brownian character of the domains in the “dynamic disordered” category are evident in dynamic fluctuations in the CV, shown in Fig. 2E for the five vesicles. The very different persistent character of the patterns in the vesicle-encompassing lattice and the closely associated configurations is evident in the analysis and reflects what is obvious to the eye when viewing the patterns live on the microscope.

### Vesicle inflation and excess membrane area

The impact of osmotic conditioning to inflate vesicles and shift the distribution of domain patterning in populations of vesicles in Fig. 1D motivates quantification of the excess area *A*_xs_. *A*_xs_ is defined as the vesicle membrane area normalized on the area a sphere of equal volume (25, 27). Therefore,


(2) $$\;A_{\mathrm{xs}}=\frac{A}{{\left(4\pi \right)}^{\frac{1}{3}}\times {\left(3V\right)}^{\frac{2}{3}}}.$$

While *A*_xs_ is related to reduced volume employed in other works (28–30), $v_{r}=\;A_{\mathrm{xs}}^{-3/2}$, focusing on *A*_xs_ emphasizes physical meaning at the level of the membrane. *A*_xs_ is measured, as described in the Supplementary material, by aspirating vesicles at low suctions into micropipettes, enabling both their volume and surface area to be quantified. Like areal strain and bending modulus measurements (6), *A*_xs_ can easily be measured to three decimal places, and often precision in the fourth decimal place is achieved (25, 31), for small micropipettes and intermediate values of the projection in the pipette that do not cause instabilities, the latter can compromise measurements above *A*_xs_ about 1.15, not critical to the current work. Sources of error are discussed in the Supplementary material. Images for low-suction pressure aspiration of vesicles exhibiting the three classes of colloid arrangements are summarized in Fig. 2A–C, along with, in Fig. 2D, a summary plot illustrating the relationship between excess area and the, CV, of the colloid arrangement prior to aspiration. This plot includes 143 vesicles studied by image analysis and then by micropipette aspiration, from 7 different electroforming batches, and 15 reconditioning runs. A strong correlation of *A*_xs_ and colloid pattern reveals the highest *A*_xs_ for the vesicle-encompassing quasi-lattices and the smallest *A*_xs_ values for associated chains and ordered clusters.

Also worthy of comment, while we were not able to quantitatively analyze entire vesicles, different focal planes through vesicles exhibiting vesicle-encompassing quasi-lattices suggest complete patterning of the entire vesicle. The nearly hexagonal arrangements of domains and their regular spacing are visually striking, especially given the variety in domain numbers per vesicle. Close scrutinization does reveal limited numbers of domains with five rather than six neighbors, as would be expected for patterning on a sphere. The term quasi-lattice is employed here for rigor since it is not possible to hexagonally pattern a sphere. Nonetheless, it is clear that domains approximate a hexagonal lattice to a great extent.

The Lindemann parameter provides a measure of the fluctuations of objects in lattices relative to their mean spacing, with $\mathrm{LP}\;=\;\mathrm{CV}/\sqrt{2}$ (32). Since fluctuations grow large toward the melting point, a threshold value of the LP is often invoked as a criterion for equilibrium melting to a fluid state. Threshold values fall in the range 0.05–0.2, depending factors like interaction type and dimensionality. While there are complexities in applying the Lindemann melting criterion in 2D systems (33, 34), even discounting the effects of finite size and spherical curvature, we note that in Fig. 2D the CV resulting from the limited triangulation method suggests a melting transition at a Lindemann parameter of 0.14 or CV = 0.2 between the vesicle-encompassing quasi-lattice configuration and the dynamic disordered state. A full analysis taking into account all colloidal plates on each vesicle, beyond our imaging capabilities, is required for rigorous quantitative analysis of vesicle-encompassing quasi-lattice structure in the future. None the less, the boundary between the vesicle-encompassing quasi-lattice configuration and the dynamic disordered state is, from the perspective of the Lindemann criterion, suggestive of a melting transition.

### State space for colloidal patterns

**Fig. 3.**
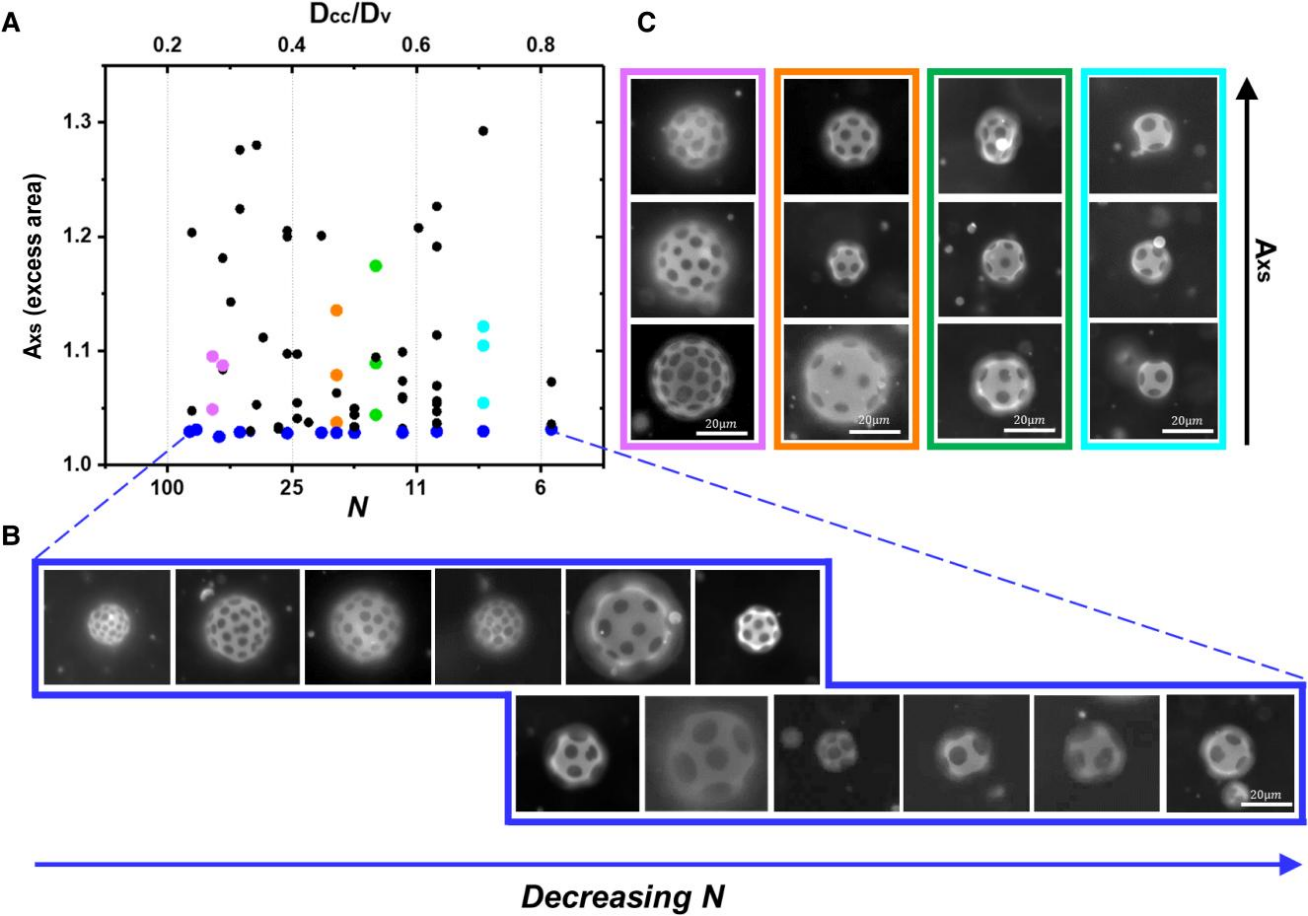
A) State space region for vesicle-encompassing quasi-lattice configurations, highlighting in blue, the vesicles with the smallest excess areas. These vesicles are shown in sequence in (B). C) Series of four vesicles each that, within each series, have similar domain number but varied excess areas. Each series is distinguished by colored points, highlighted in (A).

A potential correlation between excess area and pattern type in Fig. 2 motivates the state space of Fig. 3, with *A*_xs_ and the number of colloidal plates per vesicle, *N*, as the main parameters. When plates are uniformly distributed, as in the vesicle-encompassing quasi-lattice, in addition to the relationship in Eq. 1, *N* also relates to an approximate center–center domain spacing, *D*_cc_, normalized on the vesicle diameter, $\frac{D_{\mathrm{cc}}}{D_{v}}=\sqrt{\frac{4}{N}}$. This relationship is confirmed in the Supplementary material, for 78 vesicles that are fully encompassed by domains in a quasi-lattice configuration. The relationship persists with moderate vesicle deflation due to similar reductions in *D*_cc_ and *D*_v_ when the membrane exhibits bending fluctuations.

Figure 3A reveals that data points broadly cover the region of state space for the vesicle-encompassing quasi-lattice configuration, indicating this configuration is observed over a large range of values in *A*_xs_, *N*, and *D*_cc_/*D*_v_. Quite distinct, however, is the sharp boundary at *A*_xs_ ∼ 1.02 below which no vesicle-encompassing quasi-lattice configurations were found. Figure 3B shows example quasi-lattice structures on vesicles along the lower boundary, as the domain size is increased, or *N* is decreased at constant solid area fraction, over 1.5 orders of magnitude. Thus while the visual intricacy of the lattice pattern depends on colloidal plate size and numbers, the lattice character is preserved independent of plate size at a fixed area fraction near 17%.

Figure 3C emphasizes the minimal impact of increased excess area on overarching lattice structure, excerpting four series of different vesicles from the main plot in Fig. 3A. Within each series, the different vesicles have a fixed colloidal plate number but vary *A*_xs_. Thus it is seen that, at least near *ϕ* ∼ 0.17, for a wide range in *A*_xs_, vesicle size, and plate numbers, a vesicle-encompassing quasi-lattice is a stable if not preferred configuration. Importantly, Fig. 3A and C demonstrates the robustness of the lattice structure to vesicle dehydration, for osmotic removal of at least up to 33% of the vesicle volume, producing *A*_xs_ = 1.3. Notably, with increased deflation, vesicles may sometimes deviate from a spherical shape in favor of an irregular ellipsoid but, more generally, distributions of undulations over the entire vesicle maintain an approximately spherical global shape.

### Cooperative transition between persistent patterns

**Fig. 4.**
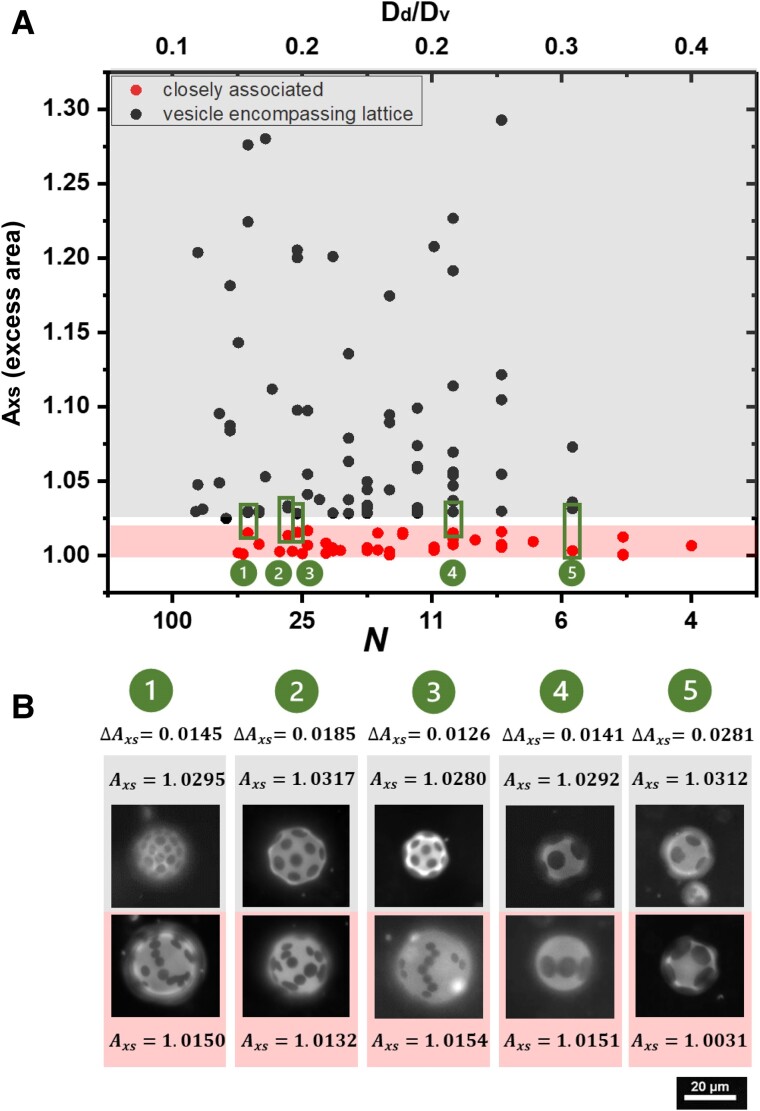
A) State space for vesicle-encompassing quasi-lattice in gray and closely associated configurations in red, with varied domain number (also translating to the domain to vesicle diamter ratio), and excess area. Five vesicle pairs, each traversing the boundary betwteen regimes, are shown in (B), highlighting the sharp morphological change of the assembly for a small change in excess area across the state space boundary.

Figure 4A expands the state space to include both types of persistent pattern classes: the vesicle-encompassing quasi-lattices and the closely associated configurations. Independent of the numbers of colloidal plates per vesicle, the vesicle-encompassing quasi-lattice is found only for *A*_xs_ > 1.025, and the associated structures are found only for *A*_xs_ < 1.015. Thus there is a sharp horizontal boundary between the two persistent pattern types. The Supplementary material highlights how vesicles with dynamic randomly positioned plates overlap these two regions, but always within the range 1.01 < *A*_xs_ < 1.05. Those vesicles containing dynamic plates may not yet have equilibrated or settled into low energy states, motivating focus on the vesicles with persistent arrangements.

Figure 4B shows the plate arrangements for pairs of vesicles highlighted in Fig. 4A, having the same plate numbers on opposing sides of the boundary separating the regimes of quasi-lattice and associated pattern types. Crossing the boundary, changing the excess area by about 1.5% profoundly alters the plate arrangement, with mostly linear configurations of closely associated colloidal plates at smaller *A*_xs_ and the vesicle-encompassing quasi-lattice at slightly greater *A*_xs_. Either type of domain arrangement includes most if not all the plates in each vesicle. The sharpness of the state space boundary suggests a cooperative assembly or a phase transition. This dramatic change in configuration for a small change in *A*_xs_ is a striking contrast to Fig. 3, where large changes in excess area above *A_xs_* ∼1.02 minimally affect the order of the vesicle-encompassing quasi-lattice arrangement.

**Fig. 5.**
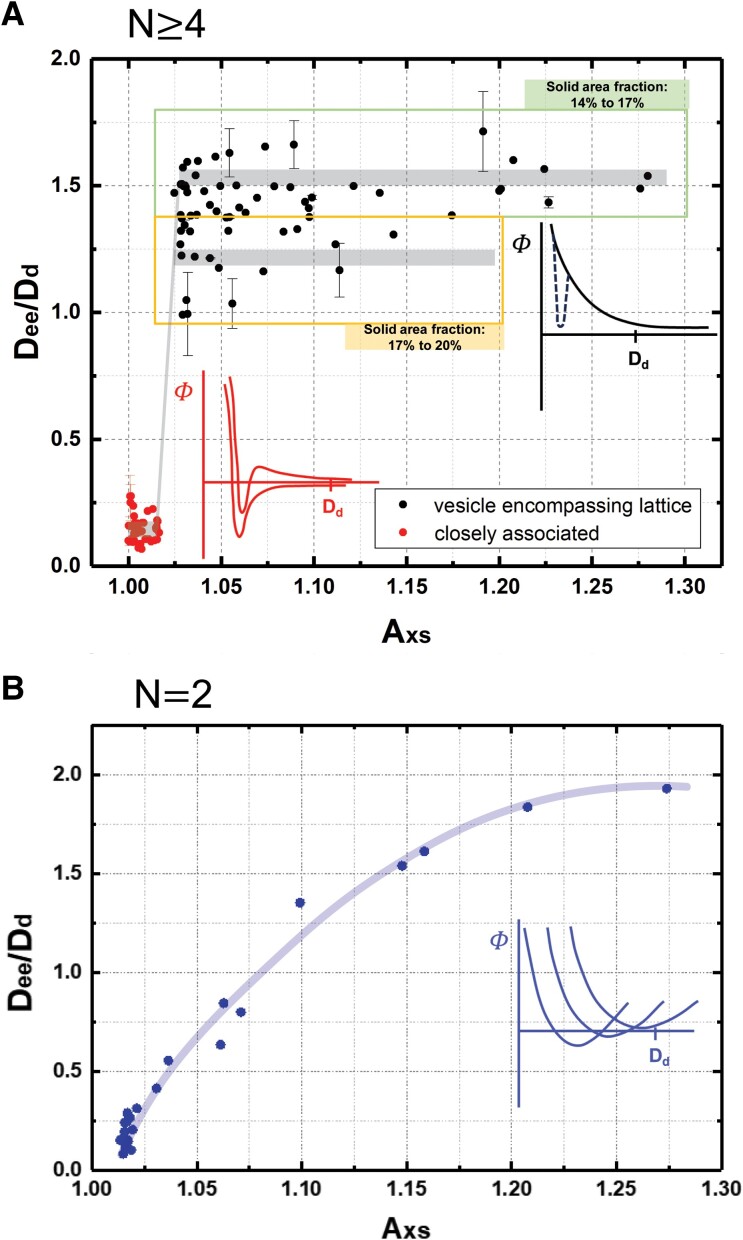
A) Normalized nearest neighbor separations, *D*_ee_/*D*_d_, measured from the edges of colloidal plates through the contoured fluid membrane at the closest points between pairs of plates and averaged, where possible, for each vesicle, then divided by the average domain diameter for that vesicle. Error bars reflect ability to resolve edge–edge separations and the impact of domain irregularity. Measurements were made within the limits of a given focal plane with resolution of ∼0.5 μm, producing larger error bars for small vesicles and small domains. Error analysis is detailed in the Supplementary material. Variation in solid area fraction indicated in (A) produces expected differences in the normalized domain separations in the vesicle-encompassing quasi-lattice. B) Edge–edge separations recalculated from prior work (25) on vesicles with two domains per vesicle and a solid area fraction in the range 15–19%.

Changes in the plate–plate interaction with variations in *A*_xs_ underlie the sharp transition in Fig. 4A and B. This is evident in Fig. 5A which summarizes the nearest neighbor edge–edge plate separations, *D*_ee_, normalized on domain or plate diameter *D*_d_, for vesicle-encompassing quasi-lattice and closely associated configurations. The plate separations are measured as the in-membrane distance between closest points on neighboring plates and averaged for plates toward the center of micrograph, where possible. Because the configurations are stable in time, the observed positioning provides a window into the effective plate–plate interaction.

Figure 5A reveals a sharp, almost step function-like transition between the close edge–edge separations at low excess area and the large edge–edge plate separations lattice pattern at slightly greater excess area and above. This sharp change in nearest neighbor separation corresponds to the crossover, in Fig. 4, from closely associated to vesicle-encompassing quasi-lattice patterns. The apparent scatter in the data for the vesicle-encompassing quasi-lattice pattern results from slight vesicle–vesicle variations in the solid area fraction that necessarily influence the edge–edge separations in a lattice. For instance, as calculated in the Supporting Information for a solid area fraction of *ϕ* = 0.19, the calculated edge–edge separation, normalized on the domain diameter is *D*_ee_/*D*_d_ = 1.18; for *ϕ* = 0.17 *D*_ee_/*D*_d_ = 1.31; and for *ϕ* = 0.15 *D*_ee_/*D*_d_ = 1.45. This fact, along with the agreement of the measured data with these calculated separations in the Supplementary material, motivated the division of the data into arbitrary subgroups that help the viewer see that the impact of variations in solid area fraction is not scatter or curvature in the data. The boxes in Fig. 5A break the data into two groups to show this behavior. Viewed differently, the sharper appearance of the step function for solid area fractions is featured in the Supplementary material.

In closely associated patterns, the edge–edge plate separations between nearest neighboring domains are similar for the different nearest neighbor pairings within the pattern of a given vesicle. The fact that the small separations are stable, without contacting of domains suggests, as drawn schematically in red, that the interaction potential contains a repulsive core and a well-defined minimum that fixes the pairwise domain positioning. The chain-like patterns suggest a longer range repulsion component of the interaction that, for groupings of three plates, pushes the third plate in an away from the first plate while still having the attractive minimum maintain associations between the first and second, and between the second and third plates. (A chain-like conformation is maintained by a repulsive interaction between the first and third plates.) This possibility is represented schematically in the same red pair potential plot as the interaction with a modest repulsion beyond the minimum.

By contrast, in the vesicle-encompassing quasi-lattice, much larger normalized edge–edge plate–plate separations are order unity, demonstrating a repulsive range on the order of the plate size or slightly larger, in the neighborhood of and sometimes exceeding 10 μm. This is illustrated schematically by the black potentials in Fig. 5A. Any attractive potential well present at high excess area is not accessible due to a substantial energy barrier, shown by the dashed line in the potential since we cannot determine whether such a feature exists. Important to note is the near-complete collapse of data when scaled on the plate diameter suggesting that the colloid size presents an important length scale in these elasticity-based interactions.

The sharp change in edge–edge plate separation occurring at $1.015\;<\;A_{\mathrm{xs}\;}<\;1.025$ in Fig. 5A for vesicles containing 5–100 domains is absent when vesicles contain only two domains. The data of Fig. 5B, recalculated from our prior work (25) by scaling on *D*_d_, reveal a more gradual increase in pairwise domain separation with *A*_xs_. Indeed the cooperative multidomain transition in vesicles with several (more than four) domains is not anticipated with vesicles containing only two domains at the same area fraction. The gradual increase in plate separation, for *N* = 2 in Fig. 5B, with *A*_xs_ is suggested schematically in the blue potentials in Fig. 5B, where a series of potentials with gradually shifting positions in their minimum is evident. This contrasts what must be a sharp change in potentials in Fig. 5A: With decreasing excess area, interactions which are entirely repulsive or which cannot access short range attractions, evolve into attractive interactions through loss of repulsions and the growth or exposure of short range attractive features.

## Discussion

How elasticity mediates colloidal interactions in interfacial systems is a topic of ongoing research. While air–water and oil–water interfaces produce capillary repulsions between objects bordered by similarly curved menisci and attractions between objects bordered by menisci with opposing local curvature, such an effect is not likely at play in the current system, which is dominated by elastic interactions rather than capillarity. The current colloidal plates are all similar within each vesicle and, while the plates within some vesicles are more sharply faceted than plates in other vesicles, no inversion of curvature was evident in the fluid near the plates. Previous modeling (25) suggests that because a rigid solid pushes Gaussian curvature into the membrane fluid, and because this curvature tends to distribute analogous to a depletion potential to minimize bending costs, the fluid acts like a spring to push domains apart. With a reduction in excess area, the distribution of curved membrane between neighboring domains adjusts to produce an attraction at a finite distance (25).

It is fascinating that, in Fig. 5A, with multiple colloidal plates interacting within a single vesicle, attractive interactions develop and dominate configurations over an extremely small range in *A*_xs_ as it is decreased toward unity and the vesicles inflate to approach a spherical shape. This scenario is reminiscent of the impact of reduced volume on the morphologies of lipids crystallizing in vesicles with different degrees of inflation (30). Bending costs become sharply prohibitive for highly inflated vesicles, above $v_{r}∼\;0.98,\;$ corresponding to *A*_xs_ in the range 1.01–1.02, forcing the growing crystals to adopt petals that bend and wrap the vesicles. A potentially related wrapping behavior is seen here in the chains of plates that encircle vesicles at their widest parts, producing a composite shape that bends globally out of plane. This reduction in energy by the bending of an elongated structure, then, is one explanation for the preference for chained configurations. An important distinction between the chained structures observed here and chains of proteins (20, 35) or nanoparticles (7, 13) in certain experiments and simulations is that molecular curvature and interactions between proteins are key in the latter (2, 3, 17, 20). Such molecular interactions are absent here and consistent with the larger finite plate separation within chains of plates.

The ability to form a single chain is limited by the numbers and size of plates in each vesicle. With membrane domains approximated as flat circular plates and when plate number *N* > $\pi \frac{D_{v}}{D_{d}},$ there are more plates than can encircle the vesicle in a single belt. This can occur at a fixed solid area fraction *ϕ* when many membrane domains nucleate in the initial formation of the colloidal plates. Then, the additional plates must branch or form a second belt, or a plaque of closely associated or ordered plates may be preferred. Observations of locally ordered clusters of plates, with vacant fluid regions elsewhere in the membrane, as in the first micrograph of Fig. 1C, suggest a reduction in the long-range repulsion that favors chain formation.

The abrupt transition in configuration (Fig. 4) and plate spacing (Fig. 5A) with excess area for vesicles containing multiple colloidal plates presents a sharp contrast to the more gradual impact of excess area in vesicles containing only two plates in Fig. 5B. It is unclear if the difference is simply that two-body interaction are always weaker when vesicle contain only two plates or if a first pair in vesicles with many domains shifts the entire interactive landscape. The considerably sharper change in plate separation with excess area in vesicles with many domains suggests a cooperative effect or phase transition.

This work focused on a single vesicle composition based on the lipid composition of 40/60 DPPC/DOPC molar ratio, giving a solid area fraction near 17%, with some variations based on temperature or loss of fluid membrane in a dislodged tether. The composition was fortuitous in revealing the rich behavior reported here. One might naturally expect variations in solid area will shift the state space or phase diagram. Exploring the impact of solid area fraction, a substantial undertaking, is an important future endeavor.

In a final note, we emphasize that multibody configurations seen here are long lived, suggesting at least meta-stability if not equilibrium. The lowest energy configurations for systems of plate-shaped colloids in 2D fluids will be further explored in future theoretical and experimental works.

## Summary and outlook

The elasticity-mediated interactions and organization of micron-scale membrane inclusions holds great potential for a variety of applications requiring flexible material sheets. These fluid–solid composites may someday provide critical functionality in applications requiring a variety of curved shapes, beyond the usual cylinders and cones of zero Gaussian curvature. The current work demonstrates the robustness of the inclusion arrangements to deflation of giant vesicles, which imposes a nonspherical shape to the sheet. The newly discovered sharp structural transition between classes of configurations within a narrow window of state space, with just a percent change in inflation or excess area, opens the door to switchable optics that respond to the slightest touch. Indeed the ability to achieve different patterns with a single material composition has important implications for using these strategies one step of the manufacturing sequence for of patterned films. At the same time, this work provides insights into the interactions of rigid membrane domains, relevant to cell biology, separate from biomolecular and physio-chemical contributions. While the domains in the current work are distinguished by their internal crystalline order, their outstanding feature is their flat, mechanically solid character that prevents substantial in-plane shear deformations. Similarly rigid features on membranes in cells might be expected to experience similar long-range elastic forces and organizational tendencies.

## Materials and methods

### Materials

l,2-dipalmitoyl-*sn*-glycero-3-phosphocholine (DPPC) and l,2-dioleoyl-*sn* -glycero-3-phosphocholine (DOPC) were purchased from Avanti Polar Lipids (catalog numbers 850375C and 850355C). A tracer lipid, Rh-DOPE 1,2-dioleoyl-*sn*-glycero-3-phosphoethanolamine-N-(lissamine rhodamine B sulfonyl) ammonium salt, catalog number 810150C, was also purchased from Avanti Polar Lipids.

### Vesicles

Giant unilamellar vesicles were electroformed on platinum wires following established procedures (36, 37). Chloroform solutions of phospholipids in the desired proportions (40 mol% DPPC, 60 mol% DOPC), at an overall concentration near 25 mg/mL, and including no more than 0.2 lipid mol% (based on the lipids) of the Rh-DOPE tracer were deposited dropwise onto the wires and dried under nitrogen. The chamber was sealed between glass coverslips, the preheated 100 mOsm sucrose solution was introduced, and the chamber was maintained near 60 °C as alternating current was applied at 3V and 10 Hz for an hour. Electroforming at elevated temperature in the one phase region of the phase diagram ensured compositional uniformity of the membranes of all vesicles, while harvesting in a syringe brought the suspension to room temperature. The 40/60 molar proportion of DPPC and DOPC produces vesicles with an expected solid domain area fraction of 17 ± 3%, based on the phase diagram, with calculations in the Supplementary material. Experimental sources error in achieving this targeted solid area fraction arises from temperature variations in the lab.

To produce vesicles containing relatively monodisperse domains, suspensions were reheated to 52 °C and maintained at this temperature for 15 min to ensure complete melting of solid domains. Subsequently, the suspension was cooled at a rate of 0.3 °C/min to 42 °C, still within the one phase region, and the temperature stabilized for 5 min. Then the system was cooled to room temperature at rates in the range 0.25–1 °C/min, with faster cooling rates selected to produce greater numbers of nucleated domains (38). Different processing runs at these different cooling rates were needed to produce the range of domain numbers in this work, varying over an order of magnitude. Experimental determination of the solid area fraction was done manually from epifluorescence micrographs, by determining the area of domains toward the center of the image (those best resolved) and multiplying the per-domain area by the numbers of domains. Error arises from the resolution of domain edges, irregularities in domain shape, and modest domain polydispersity.

### Micropipette manipulation

Excess area was assessed employing micropipette manipulation, with our apparatus described in detail previously (39, 40). Micropipettes were pulled on a Kopf Instruments micropipette puller and then forged on a Technical Products International microforge, to select the tip size and shape with nearly constant diameters in the range of 3–6 μm near the tip. Suction was applied manually using a home-built suction manometer.

### Measurements

All vesicle images were obtained using a Nikon Diaphot TE-300 inverted fluorescence microscope. Unless otherwise noted, measurements of plate and vesicle diameters were made manually using Nikon software, with a resolution as high as 1–2 pixels, at 0.34 μm/pixel, unless otherwise noted. Where possible measurements of domains and their separations were confined to the central region of vesicles, but a curvature correction in the Supplementary material was applied as needed. Also, when vesicles had small excess areas, the membrane was taut, reducing the uncertainty in edge–edge separation measurements. At large excess areas, edge–edge separation between plates, even toward the center of vesicle image, carried error due to a component of the membrane contour perpendicular to the viewing plane. These edge–edge values were corrected also as described in the Supplementary material.

Following electroforming and reprocessing in 100 mOsm sucrose, vesicles were osmotically conditioned to produce different patterns. In the studies of Fig. 1, stock vesicle solutions reprocessed in 100 mOsm sucrose were diluted by half in DI water producing 50 mOsm sucrose on the vesicle exterior. Water driven into the vesicles in these studies caused the membrane to become tense. Vesicles were imaged after 15–20 min, before there was substantial vesicle bursting. Control studied employed dilutions of the original stock suspension in glucose solution, to give a 50–50 glucose–sucrose solution on the vesicle exterior with neutral osmotic conditions of 100 mOsm.

In the studies of Figs. 2–5, stock vesicle suspensions were mixed in a 1:10 ratio with sucrose solutions having concentrations in the range 50–120 mOsm, to produce varying degrees of vesicle inflation or deflation. Vesicles were imaged after equilibration, 15 min or longer, in an open chamber that allowed micropipette manipulation. Excess area measurements were conducted by aspirating the vesicle gently into a pipette and then reducing the suction to hold the vesicle in the micropipette without stretching the membrane. From an image at this point, the vesicle area and volume were quantified (detailed in the Supplementary material), and excess area calculated via Eq. 2. It should be noted that such aspiration, especially when vesicles was first drawn into the pipette, tended to disrupt any patterning. Images of patterns were recorded prior to the aspiration of a given each vesicle studied.

## Supplementary Material

pgae331_Supplementary_Data

## Data Availability

Raw images of each vesicle employed state space and domain position analyses have been deposited in the Scholar works database (41).

## Preprints

The originally submitted version of this manuscript was posted on a preprint server: https://arxiv.org/abs/2402.15068/.
