# Supplementary material for: Bending-driven patterning of solid inclusions in lipid membranes: Colloidal assembly and transitions in elastic 2D fluids
Source: PNAS Nexus. 2024 Aug 7;3(8):pgae331. doi: 10.1093/pnasnexus/pgae331 (PMC11358708; doi:10.1093/pnasnexus/pgae331)
Supplement: pgae331_Supplementary_Data [file pgae331_supplementary_data.pdf]

Supplemental Information

Bending-Driven Patterning of Solid Inclusions in Lipid Membranes: Colloidal Assembly and Transitions in Elastic 2D Fluids

Weiyue Xin,[1] and Maria M. Santore[2]*

1. Department of Chemical Engineering
University of Massachusetts Amherst
686 N Pleasant Street
Amherst, MA 01003 United States

2. Department of Polymer Science and Engineering
University of Massachusetts Amherst
120 Governors Drive
Amherst, MA 01003 United States
santore@mail.pse.umass.edu

1. Estimating the solid area fraction from the phase diagram

We consider a membrane consisting of one mole total of DPPC and DOPC lipids. The membrane partitions into fluid (liquid) and solid domains containing L, and S moles, respectively, such that $L + S = 1$. That is, L is the moles of DOPC and DPPC taken together in the fluid membrane phase ,and S is the moles of DOPC and DPPC, taken tighter, in the solid membrane phase.

The overall mole fraction of DPPC in the entire vesicle is z_{DPPC}, and the mole fractions of DPPC in the liquid and solid phases are x_{DPPC} and y_{DPPC}, respectively.

A mass balance on the DPPC can be rearranged to give the form of the inverse lever arm rule for the amount of solid, given the composition, x_{DPPC}, y_{DPPC}, and z_{DPPC} on a tie line:

$$S = \frac{z_{DPPC} - x_{DPPC}}{y_{DPPC} - x_{DPPC}} \quad (1)$$

In equation 1, the solid is quantified in terms of S, the molar proportion of solid relative to the total moles in the entire membrane.

In order to obtain the solid area fraction, ϕ, one must include the molar areas of the fluid and solid domains, $\underline{A}_L$ and $\underline{A}_s$, respectively. Their ratio, R, is $R = \underline{A}_S/\underline{A}_L$. Then

$$\phi = \left[1 + \frac{1}{R}\frac{(y_{DPPC} - z_{DPPC})}{(z_{DPPC} - x_{DPPC})}\right]^{-1} \quad (2)$$

At room temperature, the molecular area of the fluid is approximated by the area of pure DOPC in the L_α phase, since the fluid membrane is mostly DOPC. The molecular area of DOPC in the L_α phase at 30°C is reported as 72.5A.[1] Then, using a coefficient of thermal expansion of 0.003/°C also based on the work of Nagel and Tristram-Nagel,[1] the molecular area at room temperature is found: $A_L(\text{DOPC @ 22°C}) = A_L(\text{DOPC@ 30°C})\ e^{\kappa \Delta T} = 70.8\ \text{Å}^2$.

The solid molecular area is estimated based on the $P_{\beta'}$ phase of DPPC at room temperature,[2,3] which is established to be nearly pure[4,5] such that $y_{DPPC} \sim 1$. The area of a DPPC molecule in the solid phase is estimated by starting with a value measured at 50°C in the L_α phase, $A_L(\text{DPPC @ 50°C}) = 64.0\ \text{Å}^2$.[1] From here, the footprint of DPPC in solid phase at room temperature is calculated in three steps: cooling from 50°C to T_m ~ 42°C using a coefficient of thermal expansion in the range κ = 0.003 – 0.006 /°C;[6] a 17% reduction in area for the phase transition from L_α to $P_{\beta'}$ at 41-42°C;[7] and cooling of the solid to 22°C using a coefficient of

thermal expansion in the range κ = 0.003 – 0.006 /°C. [6] This gives a range for the solid molecular area of A_S(DPPC@ 22°C) = 45.2 – 49.0 Å^2.

Finally in applying equation 2 to estimate ϕ, a composition of x_L(DPPC) = 0.18, was estimated for the end of the room temperature tie line, based on measurements of the liquidus curve of the phase diagram, by cooling vesicles having 20 mol% DPPC, and noting the temperature where solid domains first appeared.

Application of equation 2 therefore gives, for membranes containing 40 mol% DPPC, a solid fraction of:

$$\Phi = (1 + \frac{70.8}{47} * \frac{0.4 - 1.0}{0.18 - 0.4})^{-1} = 0.19$$

By considering different estimates for the coefficient of thermal expansion and compositions at the ends of the tie line we arrive at $\phi = 0.19 \pm 0.03$.

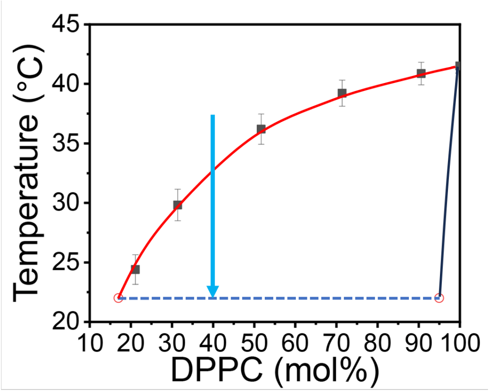

Figure S1. Phase Diagram for DOPC/DPPC mixed membranes, reproduced from previous work our this lab.[8]

2. Limited Delaunay Triangulation.

A limited version of Delaunay Triangulation, a type of pattern analysis, was pursued as a means to validate the by-eye categorization of colloidal patterns into the three types. The term “limited” is employed because a more detailed and complete use of the method, taking into account the curvature of the vesicle and including all domains in 3D, might provide further information into the nature of patterning of closed or sphere-like objects. The current work, however, cannot identify all domains on each vesicle and does not attempt geometrical transformations to account for the pseudo-spherical vesicle contour. By selecting only a subset of domains on each vesicle, the “limited” method avoids much image analysis error, at the expense of rigorous pattern analysis. The current exercise using Delaunay triangulation in a limited fashion simply aims to demonstrate that the by-eye classification of patterned vesicles can be done without bias.

Delaunay triangulation is a method in computational geometry that connects a given set of points on a plane into triangles, chosen so as to maximize the minimum angle of all the triangles in the triangulation and to allow no point in the dataset to exist inside the circumcircle of any triangle. This means that in the Delaunay triangulation, not every pair of points will be directly connected, and the edges of each triangle do not intersect.

The analysis of each vesicle commenced with the measurement of the vesicle diameter from an equatorial view and, from a top or bottom view, the identification of individual dark domains and their center points. Next, choosing the domain closest to the center of the vesicle by eye, the six closest domains to the first were identified. Then, the Delaunay-Voronoi plugin available in the FIJI software was utilized to construct the triangulation, with the triangle sides connecting the

domain centers. Then, FIJI calculated the lengths of the sides all the triangles, which provided a list of center-center domain distances based on the central and up to 6 neighboring domains.

With the idea that a regular grid of domains would have nearly identical domain spacings but elaborate patterns might be evident in other domain spacings, we calculated a coefficient of variation, a CV, taking the standard deviation of the domain spacings on a given vesicle image and normalizing by the mean.

$$CV = \frac{standard\ deviation\ of\ center - center\ spacing}{Mean\ center - center\ domain\ spacing}$$

For a perfectly ordered system, all the domains would be regularly spaced and the standard deviation and therefore the CV would vanish. An irregular grid would be expected to have a finite but small CV, and different patterns might have different CV s.

Detailed examples of Delaunay triangulation patterns are shown in Figure S2 for arbitrary domain arrays while Figure S3 shows closeups of the triangulations from Figure 1 A-C.

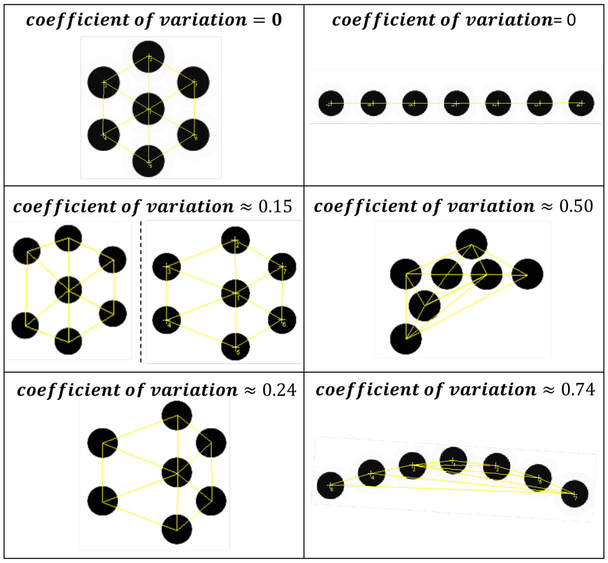

Figure S2. Example domain arrangements, their triangulation patters and coefficients of variation.

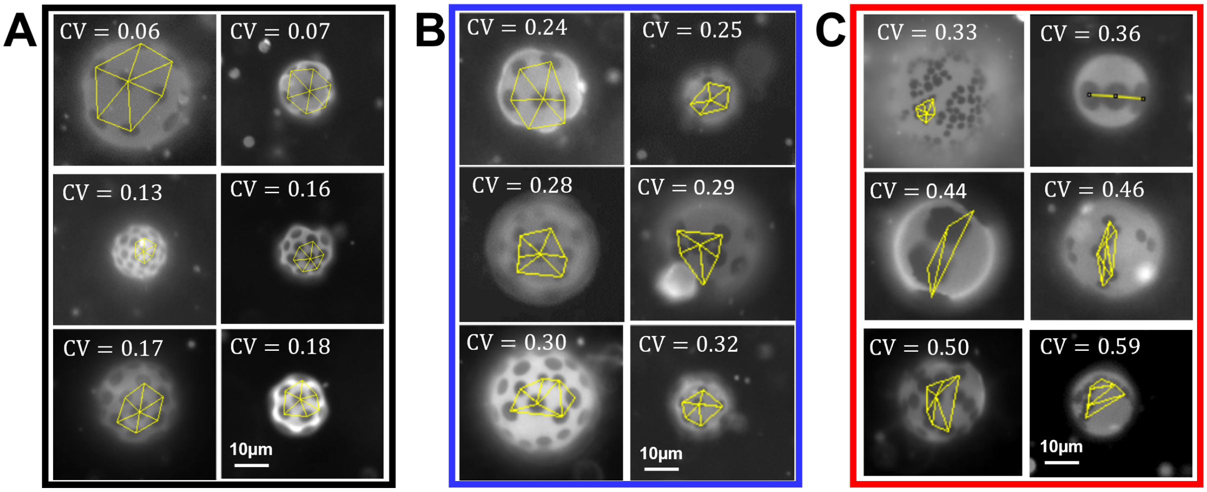

Figure S3. Enlargement of insets from Figure 1A-C showing Delaunay triangulation for example patterns on vesicles. A) vesicle-encompassing lattices B) dynamic disorder and C) closely associated.

The error in Coefficient of Variation (*CV*) can be attributed to two main factors:

Positions of domain centers: Measurement error or resolution constraints of ~2 pixels in the position of the domain center corresponds to as much as 15% error in the calculation of a single center-center domain separation. Typically the error is much less than this, just a few percent, for large domains and large absolute separations. The 15% worst case scenario occurs on small vesicles with many domains where the triangle edges are short (~5 μm) and push the limits of our resolution. However, because the lengths of all the triangle sides area not independent, the ***average*** separation length, for instance for a regular array, exhibits far less error than 15%, even for the worst case. Mis-identifying the center positions more directly impacts *CV*, by increasing the standard deviations of the triangular edges resulting from a pattern on small vesicles with many domains. This is seen, in a worst case way, by the calculated *CV* values for the hex arrays in the above table. Even with this error in *CV*, the clustering of *CV* by pattern type is clear and outside this error.

2D Projection of 3D Structures: The *CV* values ascertained by FIJI are based on 2D micrographs and do not correct for the curvature of pseudo-spherical vesicles. The impact on the measured center-center distance is small for domain pairs towards the vesicle center but more significant when the line between the centers of a domain pair has a substantial radial component. Then the radial component of the center-center line appears shorter, and domains are reported as being closer than they really are. This error does not occur for the theta component of center-center vectors between neighboring domains. For instance, the actual arc length along the membrane in the radial direction is $R\ sin^{-1}\frac{mesaured\ radial\ component}{R}$. The worst case for this type of error therefore occurs when the line connecting neighboring domains is entirely radial, with no theta

component. Then, for instance, if the center-center line starts at the middle of the vesicle and ends three quarter the distance from the center to the edge image, then center-center domain distance will be appear to be about 88% of the actual in-membrane contour between the two centers. For this reason, Delaunay analysis employed domains positioned more towards the centers of vesicles, so that error was only a few % in many cases.

3. Micropipette Measurements to Determine A_{xs}

The excess area, A_{xs}, is a dimensionless measure of the vesicle membrane area relative to that needed to encapsulate the vesicle's contents as a sphere. Increased excess area allows the vesicle to take on a variety of shapes (without stretching), or for the membrane to undulate and bend locally. A_{xs} is calculated by dividing the actual membrane area of the vesicle, A, by the area A' required to envelop the vesicle's contents in a spherical shape. When excess area equals 1, the vesicle is a sphere. Any other shape will produce an excess area exceeding 1.

For a vesicle of volume V, the radius of an equivalent sphere is

$$r' = \left(\frac{3V}{4\pi}\right)^{\frac{1}{3}} \quad . \tag{3}$$

Then the equivalent area needed to encapsulate this volume is

$$A' = 4\pi r'^2 = 4\pi * \left(\frac{3V}{4\pi}\right)^{\frac{2}{3}} \tag{4}$$

The excess area is the ratio of the actual vesicle area

$$A_{xs} = \frac{A}{A'} = \frac{A}{(4\pi)^{\frac{1}{3}} * (3V)^{\frac{2}{3}}} \tag{5}$$

Micropipette aspiration at low suction pressure to ensure membrane strain below 0.1%, was utilized to measure A and V, and then A_{xs} was calculated for each vesicle from equation 5, which corresponds to equation 2 of the main paper.

A partially-deflated vesicle, when aspirated into a micropipette forms a predominantly cylindrical projection within the micropipette and a bulbous shape outside of it. The bulb outside the micropipette takes a spherical shape except for flat solid domains. For vesicles containing ~ten to a hundred small domains, the membrane often approaches a uniform distribution of domains between the portion of the vesicle inside and external to the pipette. For vesicles containing 2-5 relatively large domains, only the fluid membrane is typically drawn into the micropipette.

Depending on a vesicle's excess area, there may (in Figure S4A) or may not be (in Figure S4B) sufficient membrane to fully establish a projection inside the micropipette at low suction. This distinction leads to the different analysis approaches, below, to estimate the vesicles' area and volume from microvideo images.

After vesicle aspiration, we measured two parameters: D_{asp}, the diameter of the vesicle outside the pipette (neglecting the impact of solid domains on its otherwise spherical contour), and L, the entire length of the projection inside the micropipette, including its cap. The pipette's inner diameter near the tip, D_p, is measured in advance. From these values, we calculate A and V. For vesicles with solid domains, we also measure their diameters, denoted D_d.

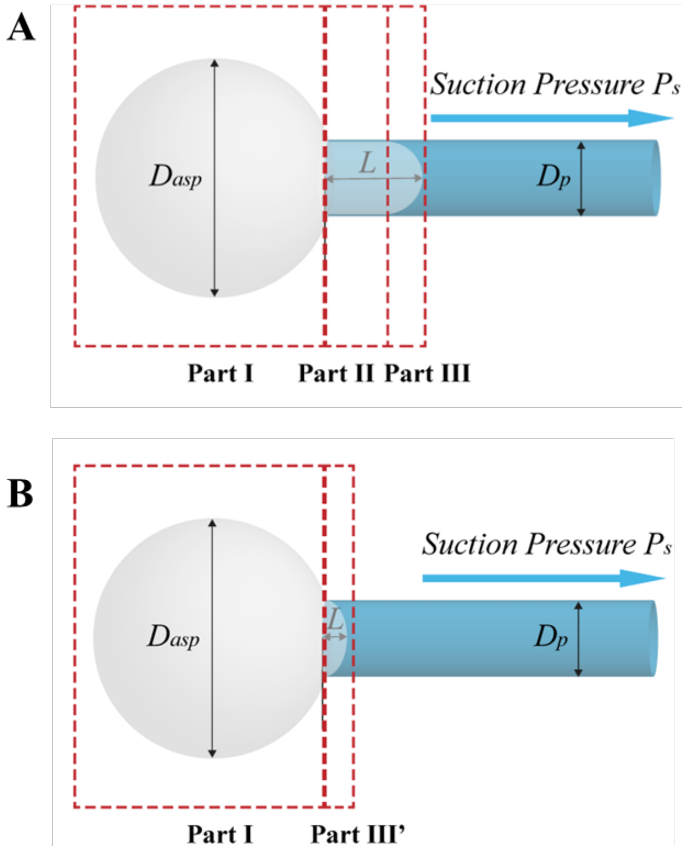

Figure S4. Shapes of vesicles aspirated into a micropipette for A) deflated vesicle producing a complete projection, and B) a vesicle with small excess area, barely entering the micropipette
Calculation of *A* and *V* is accomplished by considering parts of the vesicle which are shaped either as truncated spheres, Parts *I*, *III*, and *III'*, or as a cylinder, Part *II*. When vesicles have substantial projections, then $A = A_I + A_{II} + A_{III}$ and $V = V_I + V_{II} + V_{III}$. When the vesicle is minimally aspirated such that $L < D_P/2$ then $A = A_I + A_{III'}$ and $V = V_I + V_{III'}$. Formulas for the various vesicle parts are given in Table S1.

Table S1: Expressions for Areas and Volumes of Vesicle Parts

Part I	$A_I = \pi {D_{asp}}^2 - \pi D_{asp} * h$ $V_I = \frac{1}{6}\pi {D_{asp}}^3 - \pi h^2 (\frac{D_{asp}}{2} - \frac{h}{3})$ $h = \frac{D_{asp}}{2} - \frac{1}{2}\sqrt{{D_{asp}}^2 - {D_p}^2}$
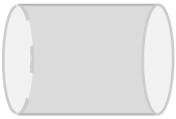 Part II $\left(L > \frac{D_P}{2}\right)$	$A_{II} = \pi D_p * (L - \frac{D_P}{2})$ $V_{II} = \frac{1}{4}\pi {D_p}^2 * (L - \frac{D_P}{2})$
Part III $\left(L > \frac{D_P}{2}\right)$	$A_{III} = \frac{1}{2}\pi {D_p}^2$ $V_{III} = \frac{1}{12}\pi {D_p}^3$
Part III' $\left(L \leq \frac{D_P}{2}\right)$	$A_{III'} = 2\pi * \left(\frac{L}{2} + \frac{{D_p}^2}{8L}\right) * L$ $V_{III'} = \pi L^2 * \left(\frac{L}{6} + \frac{{D_p}^2}{8L}\right)$

Error Analysis:

Error in determination of the excess area can arise from the of resolution in the measured parameters, D_{asp}, L, and D_p, up to ~ 2 pixels. This can produce up to 2.5% error in the determination of A_{xs}, with the worst case for small vesicles and large diameter micropipettes, for instance a 15um diameter vesicles and a 6 µm micropipette. Working at low suction maintains

the areal strain of the membrane below 0.1%, another source of error which is extremely small, and systematic across all measurements. Finally, The solid domains exist as flat plates integrated in the fluid membrane, producing deviations between the spherical treatment of the vesicle bulb outside the pipette its actual shape. Solid domains within the projection tend to bend cylindrically, not altering the projection shape and not contributing to error. The error from the flatness of the colloidal plates on the spherical bulb region depends on their number, and becomes extremely small for large N. Since the area and volume of the bulb part of the aspirated vesicle are a variable portion of the overall area and volume, the impact of error in the area and volume in the bulb on the overall error is variable, and sets an upper limit on this type of error, shown in Figure S5.

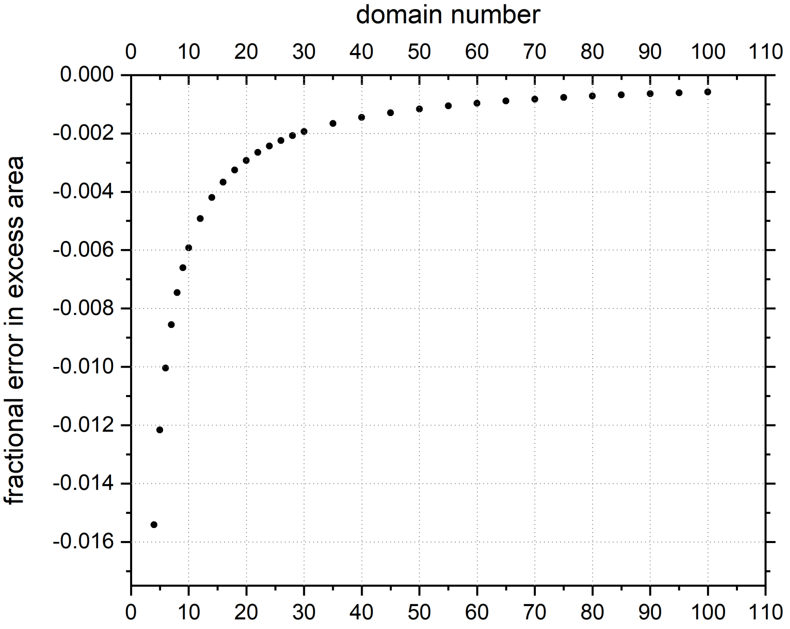

Figure S5. Fractional error in A_{xs} (an upper limit) resulting from the treatment of the bulb portion of the aspirated vesicle as a sphere, neglecting the flat plate domains. The calculation assumes aa fixed solid area fraction of 17% as N changes.

4. Center-center and edge-edge domain spacing for vesicle encompassing lattice

When N domains are roughly evenly distributed on a sphere, it is possible to estimate the center-center domain spacing, D_{cc}, by dividing the entire sphere surface into N sections, in Figure S6.

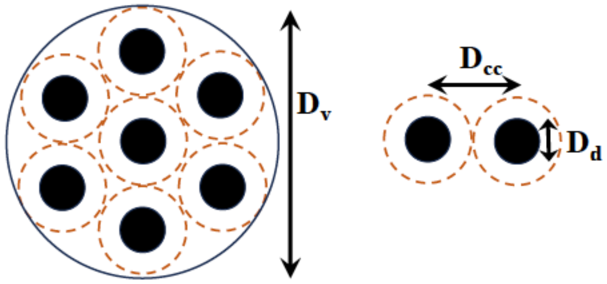

Figure S6. Schematic of domains on a vesicle, vesicle diameter, domain diameter, and center-center domain spacing. The domains at the edge of the image area not shown in the schematic. In experiments they may or may not be visible depending on focal plane, vesicle size, and depth of field. In experiments, D_v is always measured in an equatorial view.

Each section is then approximated as a circle whose diameter equals the center-center domain spacing. This gives

$$\frac{D_{cc}}{D_v} = \sqrt{\frac{4}{N}} \tag{6}$$

This relationship was tested by measuring the domain spacing of 78 vesicles that were classified as containing vesicle encompassing lattices. The results, in Figure S7A show excellent agreement between experiments and equation 6 while Figure S7B confirms the inverse half scaling with N.

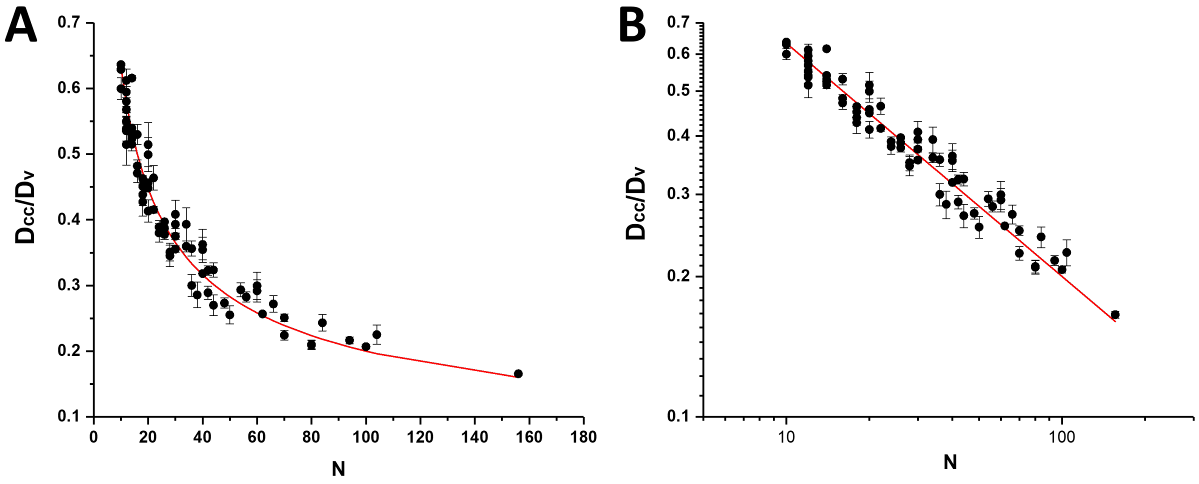

Figure S7. A. For vesicle encompassing lattices, the center-center distance measured on micrographs compared with equation 6 in red. B. Same data on log scale reveals -1/2 power scaling (red line slope.)

Figure 7A establishes that equation 6 reasonably describes normalized center-center domain spacings in the vesicle-encompassing lattice, independent of any vesicle deflation, i.e. independent of increased excess area. This is expected because if modest deflation enables membrane fluctuations, then the vesicle can appear spherical with measured vesicle diameter D_v', somewhat smaller than the fully inflated value. Then with N domains each assigned a circular membrane region, which also appears smaller than at full inflation, the center-center separation, normalized on D_v', will still follow equation 6.

Different from the center-center distances, however, the edge-edge domain separation will become disproportionately smaller with vesicle deflation. Here, the apparent and actual vesicle diameters are related by an approximate relationship: $D_v / D'_v \sim \sqrt{A_{xs}}$. While the presence of

excess area is often evident in the equatorial view, represented schematically in Figure S8, there is extra membrane between domains visualized in the top or bottom focus, even towards the center of the image where curvature in the radial direction is expected to be small. Quite often the membrane can appear bright as a result of its bending fluctuations, in addition to the curvature that is evident in the equatorial view.

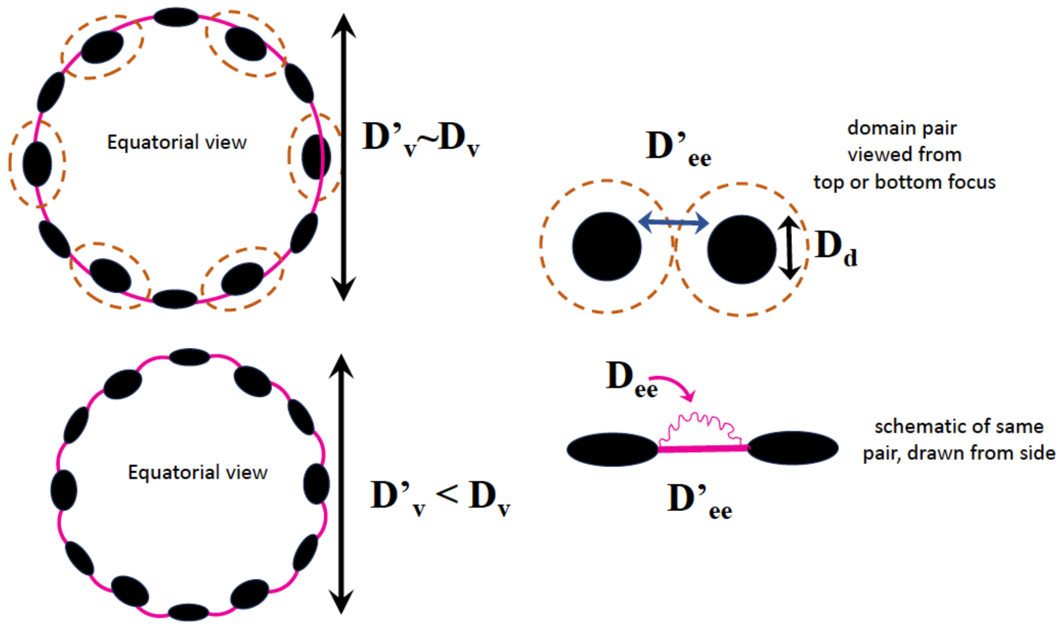

Figure S8. Schematic showing equatorial view of fully inflated vesicles and partially deflated vesicle. Illustrations with two domains show top-down view of the pair, contrasting the apparent edge-edge distance and the distance along the bending fluctuating membrane between the domain edges. Note that actual through membrane contour includes the bending that is sometimes obvious in the equatorial plane plus smaller scale fluctuations.

When we estimate the edge-edge domain separation through the membrane fluid phase the membrane fluctuations can be accounted for in an approximate fashion when the A_{xs} is quantified separately with micropipettes.

We can write for the actual fluid membrane along the circumference:

$$\pi D_v - N^{1/2} D_d. \tag{7}$$

Here D_v is the diameter of the fully inflated sphere and an approximation for the solid portion of the circumference is subtracted. Then the actual edge-edge distance between distributed domains is (dividing by $N^{1/2}$ domains in the equatorial slice)

$$D_{ee} = \frac{\pi D_v}{N^{1/2}} - D_d \tag{8}$$

And using D_v' for the apparent (measured) diameter

$$D_{ee} = \frac{\pi D'_v \sqrt{A_{xs}}}{N^{1/2}} - D_d \tag{9}$$

However, D'_{ee} is the value that is measured in a micrograph focused on the top or bottom vesicle view:

$$D'_{ee} = \frac{\pi D'_v}{N^{1/2}} - D_d \tag{10}$$

And so the apparent edge-edge separation can be corrected to include the excess membrane that bends between neighboring domains:

$$\frac{D_{ee}+D_d}{D'_{ee}+D_d} = \sqrt{A_{xs}} \tag{11}$$

Or

$$D_{ee} = \sqrt{A_{xs}}\,[D'_{ee} + D_d] - D_d \tag{12}$$

Or $$\frac{D_{ee}}{D_v} = \sqrt{A_{xs}}\left[\frac{D'_{ee}}{D_v} - 1\right] + 1$$

Note that when the excess area approaches unity, D_{ee} approaches D'_{ee}. For an apparent edge-edge separation of $\frac{D'_{ee}}{D_v} = 1.4$ the actual through-membrane separation can be as large as $\frac{D_{ee}}{D_v} =$

1.456 for large excess areas of 1.3, a worst case estimate. For a closer apparent edge-edge separation of separation $\frac{D'_{ee}}{D_v} = 1.4$, and a large excess areas of 1.3, $\frac{D_{ee}}{D_v} = 1.342$, with a slightly smaller area. Therefore the effect of excess area, domain irregularities, and imaging resolution contribute to the error in Figure 5A of the main paper. The latter two effects are more important than the impact of excess area.

5. State space including vesicles with dynamic disordered domains.

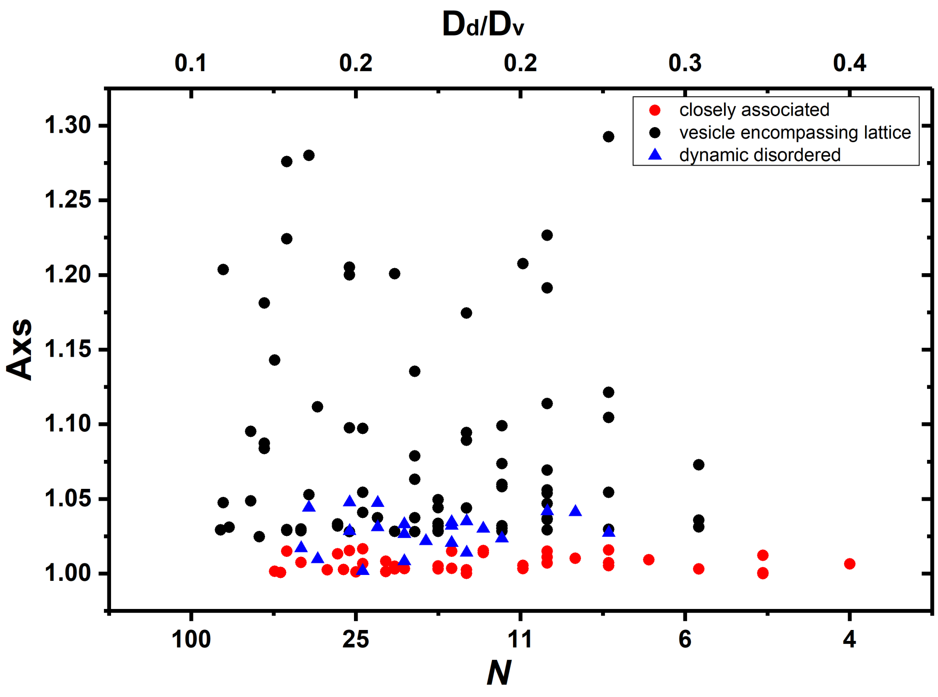

Figure S9. State space including data (blue triangles) for vesicles with dynamic disordered patterns. Vesicles having persistent domain arrangements are represented by black circles (vesicle-encompassing lattice) and red circles (closely associated configurations.)

References

1 Nagle, J. F. & Tristram-Nagle, S. Structure of lipid bilayers. *Biochimica Et Biophysica Acta-Reviews on Biomembranes* **1469**, 159-195 (2000). https://doi.org:10.1016/s0304-4157(00)00016-2

2 Chen, D. & Santore, M. M. Large effect of membrane tension on the fluid-solid phase transitions of two-component phosphatidylcholine vesicles. *Proc. Natl. Acad. Sci. U. S. A.* **111**, 179-184 (2014). https://doi.org:10.1073/pnas.1314993111

3 Chen, D. & Santore, M. M. Three dimensional (temperature-tension-composition) phase map of mixed DOPC-DPPC vesicles: Two solid phases and a fluid phase coexist on three intersecting planes. *Biochimica Et Biophysica Acta-Biomembranes* **1838**, 2788-2797 (2014). https://doi.org:10.1016/j.bbamem.2014.07.014

4 Soloviov, D. V. *et al.* in *2nd International Workshop on SANS-YuMO User Meeting at the Start-up of Scientific Experiments on the IBR-2M Reactor.* (2012).

5 Lee, S., Jeong, D. W. & Choi, M. C. Vertical order of DPPC multilayer enhanced by cholesterol-induced ripple-to-liquid ordered (LO) phase transition: Synchrotron X-ray reflectivity study. *Current Applied Physics* **17**, 392-397 (2017). https://doi.org:10.1016/j.cap.2016.12.022

6 Evans, E. & Needham, D. Physical Properties of Surfactant Bilayer Membranes - Thermal Transitions, Elasticity, Rigidity, Cohesion, and Colloidal Interactions
. *Journal of Physical Chemistry* **91**, 4219-4228 (1987). https://doi.org:10.1021/j100300a003

7 Needham, D. & Evans, E. Structure and Mechanical Propertis of Giant LIpid (DMPC) Vesicle Bilayers from 20 Degrees C below to 10 Degrees C above the Liquid Crystal Crystalline Phase Transition at 24 Degrees C. *Biochemistry* **27**, 8261-8269 (1988). https://doi.org:10.1021/bi00421a041

8 Wan, H., Jeon, G., Grason, G. M. & Santore, M. M. Flowering of Developable 2D Crystal Shapes in Closed, Fluid Membranes. *Arxiv* (2023). https://doi.org:https://doi.org/10.48550/arXiv.2307.02606
